# Total cholesterol and triglycerides are associated with the development of new bone marrow lesions in asymptomatic middle-aged women - a prospective cohort study

**DOI:** 10.1186/ar2873

**Published:** 2009-12-04

**Authors:** Miranda L Davies-Tuck, Fahad Hanna, Susan R Davis, Robin J Bell, Sonia L Davison, Anita E Wluka, Jenny Adams, Flavia M Cicuttini

**Affiliations:** 1 Department of Epidemiology and Preventive Medicine, Monash University Medical School, Alfred Hospital, 89 Commercial Road, Prahran, Victoria 3004, Australia; 2 Baker Heart Research Institute, Commercial Road, Melbourne, Victoria 3004, Australia; 3 The Women's Health Program, Department of Medicine, Monash University Medical School, Alfred Hospital, 89 Commercial Road, Prahran, Victoria 3181, Australia

## Abstract

**Introduction:**

Given the emerging evidence that osteoarthritis (OA) may have a vascular basis, the aim of this study was to determine whether serum lipids were associated with change in knee cartilage, presence of bone marrow lesions (BMLs) at baseline and the development of new BMLs over a 2-year period in a population of pain-free women in mid-life.

**Methods:**

One hundred forty-eight women 40 to 67 years old underwent magnetic resonance imaging (MRI) of their dominant knee at baseline and 2.2 (standard deviation 0.12) years later. Cartilage volume and BMLs were determined for both time points. Serum lipids were measured from a single-morning fasting blood test approximately 1.5 years prior to the MRI.

**Results:**

The incidence of BML at follow-up was associated with higher levels of total cholesterol (odds ratio [OR] 1.84, 95% confidence interval [CI] 1.01, 3.36; *P *= 0.048) and triglycerides (OR 8.4, 95% CI 1.63, 43.43; *P *= 0.01), but not high-density lipoprotein (HDL) (*P *= 0.93), low-density lipoprotein (LDL) (*P *= 0.20) or total cholesterol/HDL ratio (*P *= 0.17). No association between total cholesterol, triglycerides, HDL, LDL or total cholesterol/HDL ratio and presence of BMLs at baseline or annual change in total tibial cartilage volume was observed.

**Conclusions:**

In this study of asymptomatic middle-aged women with no clinical knee OA, serum cholesterol and triglyceride levels were associated with the incidence of BMLs over 2 years. This provides support for the hypothesis that vascular pathology may have a role in the pathogenesis of knee OA. Further work is warranted to clarify this and whether treatments aimed at reducing serum lipids may have a role in reducing the burden of knee OA.

## Introduction

The prevalence of vascular disease and cardiovascular risk factors is high amongst people with osteoarthritis (OA) [1,2]. Emerging evidence suggests that these conditions may share risk factors [1-5]. Hypercholesterolemia and hypertriglyceridemia, both risk factors for cardiovascular disease, have been related to risk of OA and the progression of OA in epidemiologic studies [1-3].

There are a number of mechanisms by which vascular pathology may contribute to the development of OA. The ends of bones are particularly susceptible to vascular insult [6]. Venous occlusion resulting in small-vessel stasis underlying the cartilage plate, joint hypertension, hypercoagulability and/or microemboli may all result in subchondral bone ischemia [3]. The resulting disturbances to subchondral bone nutrition and repair may impair the supply of nutrients and oxygen to the overlying cartilage plate [3,7,8]. Bone ischemia may also result in osteocyte death, leading to bone resorption, which may reduce the strength of the bony foundation of articular cartilage [3,8].

With the advent of magnetic resonance imaging (MRI), it is now possible to directly visualise joint structures, including cartilage and bone, in healthy subjects prior to the onset of OA. While cartilage loss is considered the hallmark of OA and is associated with symptoms [9] and risk of joint replacement [10], there is increasing evidence for a significant role of bone in the pathogenesis of knee OA and it has been suggested that bone changes may predate cartilage changes [11].

Bone marrow lesions (BMLs) are associated with knee pain [12-17] and structural changes in the knee in subjects with or without pain or radiographic OA or both. These include increased joint space narrowing [18], loss of cartilage [19-21] and cartilage defects [13,22,23]. BMLs are common in those with OA, are predominantly associated with malalignment and, once present, are unlikely to resolve [24-26]. In contrast, in asymptomatic populations [20,23], their presence is also associated with systemic factors such as dietary lipids [27] and they are more likely to resolve [28]. This is perhaps not surprising given that the histology of BML is heterogeneous and includes osteonecrosis, oedema, trabecular abnormalities and bony remodeling [29] and more recently evidence of ischemia or reperfusion injury or both [8,30]. Given the emerging evidence that OA may have a vascular basis, the aim of this study was to explore the relationship between serum lipids and (a) baseline prevalence of BMLs and (b) annual change in knee cartilage and incidence of BMLs over a 2-year period in a population of pain-free middle-aged women.

## Materials and methods

One hundred seventy-six women, 40 to 67 years old, were recruited from an existing cross-sectional study examining knee structure in women [22]. These women were initially recruited from a database established from the electoral roll in Victoria, Australia, between April 2002 and August 2003 [31]. Women were excluded if they had OA as defined by the American College of Rheumatology clinical criteria [32], current or past knee disease, a history in the past 5 years of knee pain lasting for more than 24 hours, a previous knee injury requiring non-weight-bearing treatment for more than 24 hours or surgery (including arthroscopy) or a history of any arthritis diagnosed by a medical practitioner or contraindication to MRI. The study was approved by the Alfred Hospital Human Research Ethics Committee, and all participants gave written informed consent.

### Anthropometric data and smoking status

The height and weight of each participant were measured at the time of the original study (2003 to 2005). Body mass index (BMI) was calculated from these data as weight (in kilograms) divided by height squared (in metres squared). Smoking status was determined by questionnaire.

### Measurement of blood lipids

Each participant took part in a single-morning fasting blood test at the time of the original study (2002 to 2003) approximately 1.53 years (standard deviation [SD] 0.24 years) prior to their first knee MRI. Fasting bloods drawn at the time of recruitment were stored at -80°C until assayed. Total cholesterol was determined by the CHOD-PAP (cholesterol oxidase phenol 4-aminoantipyrine peroxidase) method and triglycerides by the GPO-PAP (glycerol phosphate oxidase-p-aminophenazone) method using a Hitachi 747 analyser (Boehringer Mannheim Systems, now part of Roche Diagnostics, Basel, Switzerland). High-density lipoprotein cholesterol (HDL-C) was measured by an enzymatic colorimetric test on a Hitachi 747 analyser. The assay range is 0.1 to 20 mg/L with intra-assay coefficients of variation (CVs) of 1.34% at 0.55 mg/L and 0.28% at 12.36 mg/L, interassay CVs of 5.7% at 0.52 mg/L and 2.5% at 10.98 mg/L and a detection limit of 0.03 mg/L [33]. Low-density lipoprotein cholesterol (LDL-C) was calculated according to a method previously described [34].

### Magnetic resonance imaging and the measurement of cartilage volume and bone marrow lesion

An MRI of each woman's dominant knee (defined as the lower limb from which the subject stepped off from when initiating gait) was performed between October 2003 and August 2004 and approximately 2 years later [22]. Knees were imaged in the sagittal plane on a 1.5-T whole-body magnetic resonance unit (Philips Medical Systems, Eindhoven, The Netherlands) using a commercial transmit-receive extremity coil. The following sequence and parameters were used: fat-saturated, gradient echo, three-dimensional, T1-weighted (8 ms/12 ms/55 degrees, repetition time/echo time/flip angle, slice thickness of 1.5 mm, field of view of 16 cm and matrix of 513 × 196 pixels). In addition, a coronal, T2-weighted, fat-saturated acquisition (repetition time of 2,200 ms, echo time of 20/80 ms, slice thickness of 3 mm, 0.3 interslice gap, one excitation, field of view of 11 to 12 cm and matrix of 256 × 128 pixels) was obtained.

#### Assessment of bone marrow lesions

BMLs were defined as areas of increased signal intensity adjacent to subcortical bone present in either the medial or lateral side of the distal femur or proximal tibia assessed from coronal, T2-weighted, fat-saturated images [35]. Two trained observers, who were blinded to patient characteristics as well as the sequence of images, together assessed the presence of lesions for each subject. The presence or absence of a BML was determined. The reproducibility for determination of the BML was assessed using 60 randomly selected knee MRI scans (κ value 0.88, *P *< 0.001).

#### Cartilage volume measurement

The volumes of the individual cartilage plates (medial and lateral tibial) were measured by two blinded assessors from the total volume by manually drawing disarticulation contours around the cartilage boundaries on each section on a workstation, as previously described [9,36], at baseline and approximately 2 years later. The CVs for the medial and lateral cartilage volume measures were 3.4% and 2.0%, respectively.

### Statistical methods

Variables were assessed for normality. Age, BMI, total cholesterol, total cholesterol/HDL-C ratio and annual change in cartilage volume were all normally distributed; baseline presence and developing an incident BML compared with 'not' were binary variables. Annual change in tibial cartilage volume was calculated by subtracting the follow-up volume from the baseline volume and then dividing it by the time between MRI scans. Serum levels of triglyceride, HDL-C and LDL-C were not normally distributed and therefore the natural logarithms (ln) were used. Logistic regression was used to determine the odds of having a prevalent BML at baseline or an 'incident' BML at follow-up for each of the lipids measured. The potential confounders of age and BMI were included in the multivariate model. Linear regression was used to determine the relationship between lipids and annual change in cartilage volume. A *P *value of less than 0.05 (two-tailed) was regarded as statistically significant. All analyses were performed using the SPSS statistical package (version 15.0.0; SPSS Inc., Chicago, IL, USA).

## Results

One hundred and forty-eight (84%) of the 176 eligible women completed the 2 year follow up. Apart from being younger (*P* = 0.04) there were no significant differences in BMI (*P* = 0.31), total cholesterol (*P* = 0.85), (ln)triglycerides (*P* = 0.82), (ln)LDL (*P* = 0.38), (ln)HDL (*P* = 0.72) and total cholesterol/HDL ratio (*P* = 0.45) between those who completed the follow up and those who did not. Twenty-two (15%) of the population had a BML present in their knee at baseline. One-hundred twenty-six women were BML-free at baseline. Of them, 11 (9%) developed an incident BML over the 2-year follow-up. A comparison of baseline characteristics of the 126 women (115 who did not develop a BML and 11 who did) is presented in Table 1.

**Table 1:** Baseline characteristics of study subjects who did not develop an incident bone marrow lesion compared with those who did

	No incident BML n = 115	Incident BML n = 11	*P *value
Age, years	52.3 (6.80)^a^	54.7 (4.98)^a^	0.31^b^
Body mass index, kg/m^2^	26.9 (5.42)^b^	28.9 (5.14)^a^	0.23^b^
Total cholesterol, mmol/L^c^	5.71 (3.7-8.3)^b^	6.45 (4.8-9.1)^b^	0.04^b^
Triglycerides, mmol/L^d^	1.0 (0.5-2.9)^e^	1.4 (0.8-3.8)^e^	0.01^f^
HDL, mmol/L^c^	1.5 (0.5-2.6)^e^	1.3 (0.9-2.6)^e^	0.52^f^
LDL, mmol/L^c^	3.6 (1.8-5.8)^e^	3.87 (3.2-6.3)^e^	0.19^f^
Total/HDL ratio	3.8 (2.0-7.4)^a^	5.3 (2.5-8.3)^a^	0.13^b^

^a^Mean (standard deviation or range). ^b^Independent samples *t *test. ^c^To convert from mmol/L to mg/dL, divide by 0.0259. ^d^To convert from mmol/L to mg/dL, divide by 0.0113. ^e^Median (range). ^f^Mann-Whitney *U *test. BML, bone marrow lesion; HDL, high-density lipoprotein; LDL, low-density lipoprotein.

Serum lipids were not found to be significantly associated with the presence of BMLs at baseline (Table 2). The relationships between serum lipids and incidence of BMLs are presented in Table 3. Incident BMLs were associated with higher total cholesterol and triglyceride concentrations but not HDL-C, LDL-C or total cholesterol/HDL-C ratio. Only one woman who developed a BML had ever smoked, so the relationship between smoking and incident BML could not be examined. The odds of developing a BML were 1.84 (95% confidence interval [CI] 1.01, 3.36) for every 1 mmol/L increase in total cholesterol after adjusting for the potential confounders of age and BMI (*P *= 0.048). The odds of an incident BML were 8.4 (95% CI 1.63, 43.43) for each unit increase in (ln)triglycerides after adjusting for confounders (*P *= 0.01). A trend between increased odds of incident BMLs and total cholesterol/HDL-C was also observed (odds ratio [OR] 1.53, 95% CI 0.98 to 2.38; *P *= 0.06) in univariate analyses, but this relationship did not reach significance after adjustment for confounders (OR 1.41, 95% CI 0.86 to 2.32; *P *= 0.17). All analyses were also performed adjusting for weight rather than BMI, but this did not alter the results (data not shown).

**Table 2:** Association between bone marrow lesions at baseline and lipids

	Univariate analysis, odds ratio (95% CI)	*P *value	Multivariate analysis, odds ratio (95% CI)^a^	*P *value
Total cholesterol	0.91 (0.62, 1.33)	0.63	0.9 (0.6,1.35)	0.61
Ln triglycerides	1.99 (0.77, 5.16)	0.15	1.81 (0.63, 5.20)	0.27
Ln HDL	0.45 (0.09, 2.12)	0.31	0.63 (0.11, 3.50)	0.6
Ln LDL	0.65 (0.15, 2.90)	0.58	0.58 (0.12, 2.80)	0.50
Total/HDL ratio	1.10 (0.81, 1.50)	0.54	1.03 (0.73, 1.43)	0.88

^a^Odds ratio of having a bone marrow lesion present at baseline for each unit increase in total cholesterol, natural logarithm (Ln) of triglycerides, natural logarithm of high-density lipoprotein (HDL) or low-density lipoprotein (LDL) or for the total/HDL ration after adjusting for age and body mass index. CI, confidence interval.

**Table 3:** Association between incident bone marrow lesions and lipids

	Univariate analysis, odds ratio (95% CI)	*P *value	Multivariate analysis, odds ratio (95% CI)^a^	*P *value
Total cholesterol	1.82 (1.04, 3.2)	0.037	1.84 (1.01, 3.36)	0.048
Ln triglycerides	9.23 (2.06, 41.46)	0.004	8.4 (1.63, 43.43)	0.01
Ln HDL	0.60 (0.06, 5.99)	0.67	1.12 (0.008, 14.79)	0.93
Ln LDL	7.10 (0.56, 89.39)	0.13	5.79 (0.39,86.46)	0.20
Total/HDL ratio	1.53 (0.98, 2.38)	0.06	1.41 (0.86, 2.32)	0.17

^a^Odds ratio of having an incident bone marrow lesion for each unit increase in total cholesterol, natural logarithm (Ln) of triglycerides, natural logarithm of high-density lipoprotein (HDL) or low-density lipoprotein (LDL) or for the total/HDL ration after adjusting for age and body mass index. CI, confidence interval.

In addition, when all people who had a BML at either baseline or follow-up (n = 33) were grouped and compared with those who had never had a BML (n = 115), we found that the difference in BMI between the two groups approached significance with a trend of *P *= 0.09 (BML group: mean 28.8 mmol/L, SD 6.3; no BML group: mean 26.9 mmol/L, SD 5.4), but there was no difference in age or gender. Triglyceride levels were also significantly higher in those with a BML compared with those who did not have a BML at either time point (median 1.3 mmol/L, range 0.7 to 3.8, compared with median 1.0 mmol/L, range 0.5 to 2.9; *P *= 0.02). This persisted after adjusting for age and BMI (OR 3.28, 95% CI 1.16 to 9.21; *P *= 0.024).

No association between total cholesterol, triglycerides, HDL-C, LDL-C, total cholesterol/HDL-C ratio and smoking status and annual change in total tibial cartilage volume was observed (Table 4). Similarly, when the medial and lateral compartments were analysed separately, no association was seen (data not shown).

**Table 4:** Association between lipids and annual change in total tibial cartilage volume

	Univariate analysis, regression coefficient (95% CI)	*P *value	Multivariate analysis, regression coefficient (95% CI)^a^	*P *value
Total cholesterol	-0.24 (-12.77, 12.28)	0.97	-1.77 (-11.35, 14.88)	0.79
Ln triglycerides	0.70 (-32.48, 33.88)	0.97	12.6 (-24.12, 49.32)	0.49
Ln HDL	-4.78 (-56.43, 46.88)	0.85	-15.96 (-72.28, 40.37)	0.58
Ln LDL	3.16 (-48.06, 54.39)	0.90	13.12 (-41.2, 67.45)	0.63
Total/HDL ratio	-0.67 (-11.57, 10.24)	0.90	2.58 (-9.5, 14.68)	0.67

^a^Annual change in total tibial cartilage volume (in microlitres) for each unit increase in total cholesterol, natural logarithm (Ln) of triglycerides, natural logarithm of high-density lipoprotein (HDL) or low-density lipoprotein (LDL) or for the total/HDL ratio after adjusting for age and body mass index. CI, confidence interval.

## Discussion

In this cohort study of asymptomatic middle-aged women, serum lipids were not associated with the presence of BMLs at baseline or change in knee cartilage over 2 years. However, greater levels of total cholesterol and triglycerides, even within the normal ranges, were associated with the incidence of BMLs in knees free of BMLs at baseline.

No previous study has examined the relationship between serum lipids and longitudinal change in knee structures or incident OA. There is, however, some evidence suggesting a relationship between increasing cholesterol and knee OA from cross-sectional studies [37,38]. Among patients selected based on hospitalisation for joint replacement due to advanced OA, approximately 38% had hypercholesterolemia (serum cholesterol of at least 6.2 mmol/L or on antihyperlipidemic medications) [37]. Among women from the Chingford study, moderately raised serum cholesterol levels (6.0 to 7.1 mmol/L) were associated with the presence of radiological and bilateral knee OA [38]. In our population of asymptomatic subjects with no clinically knee OA, we found no significant relationship between serum lipids and change in cartilage over 2 years.

In contrast, we found a significant relationship between serum lipids and the development of new BMLs. This finding is supported by the recent findings in a different asymptomatic population that found that dietary lipids were associated with the risk of BMLs [27]. Whilst total cholesterol and triglycerides were associated with the incidence of BMLs, no relationship was seen with the traditional vascular risk factors of total cholesterol/HDL ratio and LDL. An increased ratio indicates a higher concentration of the more atherogenic LDL compared with the HDL cholesterol and confers an increased cardiovascular risk [39]. This may be due in part to the small number of incident BMLs reducing the power of the study to detect weaker associations, as was seen in the demonstrated relationships between incident BML and total cholesterol/HDL ratio, in which a significant univariate trend that was not persistently significant in the multivariate analyses was shown. Although we did not detect any significant associations between lipid levels and prevalent BMLs at baseline, this may be explained by the mixed nature of BMLs. To date, a number of risk factors have been identified for BMLs; one predominant risk factor is malalignment [18]. Prevalent BMLs are likely to be a diverse group and the result of a number of risk factors, including altered joint biomechanics and trauma as well as other known and unknown factors. Therefore, the ability to identify a risk factor such as serum lipids, independent of other risk factors, may be reduced among those with prevalent BMLs. In contrast, by examining those free of BMLs at baseline in this population of asymptomatic people with no clinical knee disease or symptoms, we were able to detect a relationship between serum lipids and incident BMLs. This longitudinal finding provides a much stronger level of evidence for a relationship between serum lipids and BMLs than can be obtained from a cross-sectional study [40]. Our data suggest that BMLs are not solely a consequence of biomechanical factors. It is possible that the relationship between lipids and BMLs may be a consequence of vascular pathology. Subchondral bone is highly vascularised and it has been suggested that one origin of BMLs may be ischemia and/or reperfusion injury [8]. A study of human bone marrow using gadolinium demonstrated that compared with knees free of BMLs, those with BMLs showed perfusion abnormalities, including significantly reduced venous outflow [30], and BMLs detected on MRI have been said to be similar to those seen in avascular necrosis [7].

Although some previous studies have suggested that hyperlipidemia may occur as a consequence of OA and the associated treatments, the findings of our study suggest that this is not the case but that serum cholesterol and triglycerides are positively associated with structural change in the knee in the absence of OA. Whether elevated lipid levels cause incident BMLs via a vascular mechanism is not known. Alternatively, it may be that the relationship between serum lipids and incidence of BMLs is the result of inflammatory pathways or as yet unknown mechanisms. Given that coagulation and inflammatory pathways have been shown to be intimately related [41], it is possible that the results we have observed are a combination of both effects. Histological studies in both animals and humans have demonstrated lipid, cholesterol and fibrin deposits in cancellous bone [41-43]. These lipid emboli and thrombi may result in reduced blood flow and lead to ischemia and ultimately bone necrosis. Furthermore, dogs with hip OA were shown to have increased serum lipid levels as well as evidence of hypofibrinolysis and increased platelet aggregability that could be reversed with treatment, resulting in a significant improvement in OA symptoms [41]. Thus, it may be that reducing lipids will have a beneficial effect in reducing subsequent knee OA.

Hypercholesterolemia and hypertriglyceridemia are major risk factors for cardiovascular disease in women [44]. We have found that subtle perturbations in lipid metabolism are also associated with the development of new BMLs, which are significant predictors of OA development and progression. We did not find a significant association between serum lipids and cartilage change. However, given that BMLs are associated with progression of cartilage defects [45] and loss of cartilage [19-21], it may be that our present study did not have power to show a direct relationship between serum lipids and cartilage loss. Larger studies of longer duration, especially in an asymptomatic population free of clinical OA, may be required.

This study has a number of limitations. First, the power of this study to show an effect was limited by the low number of prevalent (14%) BMLs; therefore, we were unable to examine change in BML size over 2 years. In addition, due to the modest number of subjects who developed a BML, these results will need to be confirmed in larger studies. In this study, we were not able to measure knee alignment and this may have attenuated our findings. However, recent work suggests that the relationship between BML and progression persisted after accounting for alignment [18]. In addition, due to the low prevalence of smokers in this population, we were unable to examine how smoking may relate to loss of cartilage and incidence of BMLs. We did not obtain radiographs of the knees, so some subjects may have had asymptomatic radiographic OA. However, we used the presence of pain to exclude any potential participants who may have had clinical OA since all of the American College of Rheumatology criteria for the classification of knee OA require pain. Individuals with significant knee injury in the past, pain at baseline, knee surgery or physician diagnosis of any type of arthritis were excluded. There is debate as to how to define early knee OA since it is now clear that there is a continuum from the normal to the OA knee, with more than 10% of knee cartilage already lost by the time radiological OA is present [46]. A major strength of our findings is that serum lipids were shown to be a risk factor for incident BML among those without any BML at baseline in a population that was asymptomatic with no knee pain, no history of treatment for knee disease and no history of knee injury. This population represents an asymptomatic preclinical OA population within the spectrum of disease development and would be the population in which primary prevention measures would be used. Another potential limitation of our study is that lipids were measured 1.5 to 3.5 years prior to the MRI assessment. However, baseline measurements of lipoprotein lipids and apoproteins have been shown to be robust predictors of cardiovascular events a number of years later [47]. We also did not have information on whether women were on lipid-lowering drugs; however, the aim of this study was to examine the relationship between serum lipid levels and BMLs and cartilage. Any woman on treatment would have been classified based on her cholesterol level; therefore, those with a tendency to hypercholesterolemia but whose cholesterol was normal on assessment would have been analysed as having a normal cholesterol level. This is likely to have underestimated the effect we observed.

## Conclusions

In this study of asymptomatic middle-aged women with no clinical knee OA, cholesterol and triglyceride levels were associated with the incidence of BMLs over 2 years. This provides support for the hypothesis that vascular pathology may have a role in the pathogenesis of knee OA and warrants further work to clarify this and whether treatments aimed at reducing serum lipids may have a role in reducing the burden of knee OA.

## Abbreviations

BMI: body mass index; BML: bone marrow lesion; CI: confidence interval; CV: coefficient of variation; HDL: high-density lipoprotein; HDL-C: high-density lipoprotein cholesterol; LDL: low-density lipoprotein; LDL-C: low-density lipoprotein cholesterol; ln: natural logarithm; MRI: magnetic resonance imaging; OA: osteoarthritis; OR: odds ratio; SD: standard deviation.

## Competing interests

The authors declare that they have no competing interests.

## Authors' contributions

FH, SRD, SLD and JA were involved in the design and implementation of the study, including data collection and measurement. AEW, RJB and FMC were involved in the design and implementation of the study, including data collection and measurement, and in the analysis and interpretation of the data. MD-T was involved in the analysis and interpretation of the data. All authors were involved in the manuscript preparation and read and approved the final manuscript.
